# Micronutrient Gaps and Supplement Use in a Diverse Cohort of Pregnant Women

**DOI:** 10.3390/nu15143228

**Published:** 2023-07-20

**Authors:** Sarah A. Crawford, Alexandra R. Brown, Juliana Teruel Camargo, Elizabeth H. Kerling, Susan E. Carlson, Byron J. Gajewski, Debra K. Sullivan, Christina J. Valentine

**Affiliations:** 1 Department of Dietetics and Nutrition, The University of Kansas Medical Center, Kansas City, KS 66160, USAscarlson@kumc.edu (S.E.C.); dsulliva@kumc.edu (D.K.S.); 2 Department of Biostatistics & Data Science, The University of Kansas Medical Center, Kansas City, KS 66160, USAbgajewski@kumc.edu (B.J.G.); 3 Department of Pediatrics, Division of Neonatology, Banner University Medical Center, The University of Arizona, Tucson, AZ 85719, USA

**Keywords:** pregnancy, nutrition, dietary supplements

## Abstract

Background: Micronutrition in pregnancy is critical to impact not only fetal growth and development but also long-term physical and psychiatric health outcomes. Objective: Estimate micronutrient intake from food and dietary supplements in a diverse cohort of pregnant women and compare intake to the Dietary Reference Intakes (DRIs). Design: Secondary analysis of women enrolled in a multi-site clinical trial of docosahexaenoic acid (DHA) supplementation who provided their dietary intake using the diet history questionnaire-II (*n* = 843) or multiple 24 h recalls (*n* = 178) at baseline and their intake of nutritional supplements at baseline through 30 days postpartum. Participants/Setting: 1021 participants from the parent trial who had reliable data for dietary intake, supplement intake, or both. Main outcome measures: Micronutrient intake from dietary and supplement sources and percentage of intakes meeting the DRIs for pregnancy. Statistical analyses performed: Percent of participants whose intake was below the estimated average requirement (EAR) or adequate intake (AI) and above the tolerable upper limit (UL). Results: Dietary intakes of choline, folate, iron, vitamin D, zinc, vitamin E, magnesium, and potassium, were below the AI or EAR for 30–91% of the participants; thiamin and vitamin B6 were also below the AI or EAR for non-Hispanic/Latina women. Supplement intake improved the intake for most; however, 80% of the group remained below the AI for choline and 52.5% for potassium while 30% remained below the EAR for magnesium. Folate and iron intakes were above the UL for 80% and 19%, respectively. Conclusions: Dietary supplements, despite their variability, allowed the majority of this cohort of pregnant women to achieve adequate intakes for most micronutrients. Choline, magnesium, and potassium were exceptions. Of interest, folate intake was above the tolerable UL for the majority and iron for 16.8% of the participants. Clinicians have the opportunity to address the most common nutrient deficits and limits with advice on food sources that provide choline, magnesium, and potassium and to ensure folate is not overabundant. More research is needed to determine if these findings are similar in a cross-sectional population.

## 1. Introduction

The American College of Obstetricians & Gynecologists and the Academy of Nutrition and Dietetics recommend that women consume a prenatal multivitamin with folic acid and iron before and during pregnancy in combination with a healthy diet [1,2]. Data from the National Health and Nutrition Examination Survey (NHANES), between 1999–2014, demonstrated that 90.8% of the pregnant women surveyed took at least one dietary supplement and 80.4% reported using a product identified as being for prenatal use during at least one trimester of pregnancy [3]. The first 1000 days, starting from conception to 2 years, is a crucial period for organ development that can impact lifelong cognition, behavior, psychiatric, and immune health [4]. For this reason, it is critical to understand what nutrients in the contemporary setting are inadequate in the diet and how supplements can improve micronutrient intake for the pregnant mother. Often mothers are unaware of the effect her diet and supplement use can have on this time-period.

Adding to the complexity is that the industry providing supplements in the United States (US) [5] can be quite variable in the form of the product as well as the nutrient composition. According to the latest information from the National Institutes of Health (NIH) Office of Dietary Supplements’ (ODS) Dietary Supplement Label Database (DSLD) (August 2022), there are 718 products currently on the market with the word “prenatal” in the product name or somewhere else on the label. Over 75% are multivitamin/mineral supplements [6]. The content of prenatal supplements varies greatly in both type and amounts of micronutrients, which suggests their design is not based on scientific evidence.

It is important to know where micronutrient gaps exist so that clinical providers can best educate pregnant women about the food sources and dietary supplements they should consume in pregnancy. This information is also important for companies that manufacture prenatal supplements to better design and meet existing dietary micronutrient gaps as well as to ensure that supplement intake does not result in micronutrient intakes that exceed the tolerable upper limit (UL). Unfortunately, there is a paucity of recent information on micronutrient intake by US pregnant women. Bailey et al. [7] reported the combined results of micronutrient intake from seven cycles of NHANES from 1999 to 2014; however, most women included were prior to 2004. Jun et al. [3] looked at results from the same NHANES cycles to determine how maternal age, income, and trimester of pregnancy was related to supplement use. Maternal factors such as age, education, family income, and race/ethnicity were also the subject of a study on the risk of inadequate and excessive micronutrient intake from diet and supplements in 15 observation cohorts of singleton pregnancies in the US for the Environmental Influences on Child Health Outcomes (ECHO) consortium [8]. The composition of multivitamins continues to evolve, and information from supplement intake twenty years ago may not represent current nutrient intake from supplements [9], supporting the need for a more recent evaluation of micronutrient intake from diet and supplements by pregnant women in the US. Moreover, NHANES collects supplement intake information at a single time regardless of the stage of pregnancy and asks only for intake information from the past 30 days [3,7]. Our study was conducted from 2016–2020, and we obtained detailed information on supplement intake beginning 6 months prior to pregnancy through the first 30 days postpartum to obtain a comprehensive understanding of dietary supplement use and the kind and variety of supplements consumed.

Our primary goals were to estimate the micronutrient intake from diet and supplements in a large cohort of US pregnant women, identify how all dietary supplements contribute to women achieving the Dietary Reference Intakes (DRIs) for nutrients in pregnancy, and determine if nutrient gaps exist. A secondary goal was to evaluate the relationship between demographic and social characteristics with prenatal dietary supplement use.

## 2. Materials and Methods

### 2.1. Study Design

This is a secondary analysis of diet and supplement intake that was collected prospectively for the primary aim of evaluating how micronutrient intake influenced the effect of docosahexaenoic acid (DHA) supplementation on preterm birth. The multicenter, Phase III, double-blind, randomized clinical superiority trial “The Assessment of DHA on Reducing Early Preterm Birth (ADORE)” was conducted from June 2016 to September 2020 and was supported by the Eunice Kennedy Shriver National Institute of Child Health & Human Development and the Office of Dietary Supplements (R01HD083292). Supplemental funding came from the Office of Dietary Supplements (ODS) (R01 HD083292-03S1) [10]. All 1100 participants in the trial provided written consent for the primary study and for study activities related to nutritional assessment under a central IRB (University of Kansas Medical Center, STUDY00003455). The trial was registered (ClinicalTrials.gov: NCT02626299) on 8 December 2015.

### 2.2. Participants

Participants were 18 years of age or older with a singleton pregnancy who could read and speak in English or Spanish, and only women who did not meet these criteria were excluded. The trial was conducted under as an Investigational New Drug trial (#129,482), and the FDA limited the exclusion criteria to ensure recruitment was generalizable. In brief, the group was diverse in race and ethnicity (67.5% white, 26% Black/African American, 20.7% Hispanic/Latina, 3.1% Asian, 2.7% Bi- or Multiracial, and 0.5% American Indian or Alaskan Native), education level (ranging from less than high school (14.5%) to a doctorate (8%)), and by family income (<USD 15,000, 21% to >USD 150,000, 11%). Seventy percent had a prior pregnancy (Table 1). Women were enrolled in the trial in Kansas City, KS, Cincinnati, OH, or Columbus, OH between 12- and 20-weeks’ gestation and followed through 30 days postpartum. All Spanish-speaking participants were enrolled in Kansas City by a Spanish-speaking staff member.

### 2.3. Dietary Intake

**Figure 1:**
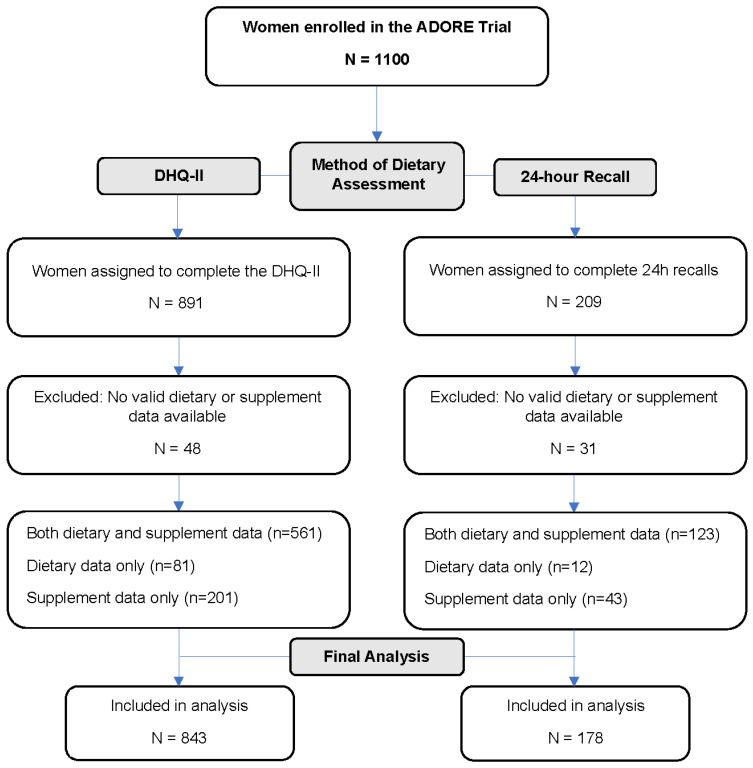
CONSORT framework.

Dietary intake was assessed at baseline using the National Cancer Institute’s (NCI) diet history questionnaire-II (DHQ-II) (*n* = 843) or three 24 h dietary recalls (*n* = 178). See Figure 1 for the consort framework. The DHQ-II is a food frequency questionnaire (FFQ) that was developed by the Risk Factor Assessment Branch of the NCI’s Epidemiology and Genomics Research Program [11]. It consists of 134 items asking about dietary intake over the past year, including questions about portion size, and it has been validated to assess overall dietary intake [12,13,14].Questionnaires were analyzed using the Nutrient and Food Group Database (released December 2014) [15] and Diet*Calc software (version 1.5.0; released October 2012) [16], which are both available for download from the NCI website [17,18]. Multiple-pass 24 h recall interviews were obtained on non-consecutive days (two weekdays and one weekend day) and were conducted by trained staff members. This method is shown to represent usual individual dietary intakes [19], using a multiple-pass approach [20]. Participants were provided with a Food Amounts Booklet in their preferred language to assist in estimating portion sizes consumed [21,22]. To reflect the marketplace throughout the study, dietary intake data were collected using the Nutrition Data System for Research (NDSR) (software versions 2016, 2017, and 2019, University of Minnesota, Minneapolis, MN); however, the final calculations were completed using NDSR version 2019 [23,24,25]. Participants were asked to complete the DHQ-II unless they identified as Hispanic/Latina individuals. Hispanic/Latina participants who spoke Spanish, and all but 33 of those who spoke English, were asked to complete three 24 h recalls. At the time the study began, [26] of the DHQ-II were not validated for the Hispanic/Latina population. Seventy-nine participants had neither complete nor valid dietary or supplement intake data. Of the remaining 1021 women, 684 had both dietary and supplement intake data available. Ninety-three women had valid dietary intake data but had missing or invalid supplement data, while 244 subjects had usable supplement intake data but missing or invalid dietary data. Data for individuals were deemed incomplete if the DHQ-II was not completed or the 24 h recalls were available. Dietary data were considered invalid if a daily intake of <1075 or >4777 kcals (<4.5 MJ or >20 MJ) [27] was recorded. Of the 843 participants asked to complete the DHQ-II, only 642 (76%) had a questionnaire that could be used, while 135 of 203 (67%) had dietary intake data from 24 h recalls.

We analyzed the intake of the following 21 micronutrients: choline, folic acid, niacin, riboflavin, thiamin, vitamin A, vitamin B6, vitamin B12, vitamin C, vitamin D, vitamin E, calcium, chromium, copper, iodine, iron, magnesium, manganese, potassium, selenium, and zinc and the supplement intake of iodine and chromium. Because the results of the DHQ-II and NDSR have not been compared, the dietary intake obtained are reported separately for both methods.

### 2.4. Supplement Intake

Information was collected on all supplements consumed from 6 months before pregnancy until enrollment and approximately every 4 to 6 weeks thereafter through 30 days postpartum. The average intake of each nutrient throughout pregnancy was added to the baseline dietary intake of the nutrient. On each occasion, the study coordinator asked about and recorded the supplement intake, including any changes such as discontinuing a supplement, starting a new supplement, or a change in the serving/dose or frequency of a current supplement (i.e., taking two capsules instead of one or taking it 5 days per week instead of 7). Changes to the dose or frequency of a supplement were tracked to obtain the best representation of the participant’s intake. Details were recorded for each unique supplement reported by a participant, including the brand and name of the supplement, start date, stop date (if applicable), dose or serving size consumed, and frequency (number of days per week) the supplement was taken. If the start and/or stop dates were unknown, staff asked probing questions to estimate the approximate month or season of intake. Participants were encouraged to bring bottles or take pictures of supplement labels so that staff could confidently identify the exact supplement.

Of the 93 participants whose supplement intake data were not included, 16 did not use a dietary supplement during their pregnancy. The remaining 77 participants reported taking one or more supplements; however, they did not provide enough detail to estimate the nutrient content of the supplement.

### 2.5. Supplement Database

We created a database that contains label information for each supplement reported. There were 472 unique supplements. Ninety-four did not contain nutrients of interest, e.g., amino acids, fiber, probiotics, and botanicals. Another 24 supplements included a nutrient of interest, but there was missing information on participant intake. The remaining 354 were included in our analyses. All supplements were categorized by the investigators based on the content, name, and primary purpose (for example, a fish oil supplement containing vitamin E was categorized in the category omega-3 fatty acid).

Of the 354 supplements analyzed, only 136 met the definition of a prenatal multivitamin–mineral supplement, i.e., containing ≥3 vitamins + ≥1 mineral and specifically marketed for conception, prenatal, or postnatal use. This is the same definition used by Bailey et al. [7]. Forty-one of the supplements were general multivitamin–mineral supplements (containing ≥3 vitamins + ≥1 mineral and not designated for conception, prenatal, or postnatal use), and 153 were single vitamin or mineral supplements (any other vitamin or mineral supplement that did not meet the definition of a prenatal or general multivitamin-mineral). The remaining 24 supplements included amino acid supplements (*n* = 2), fiber or probiotic supplements (*n* = 5), herbal or botanical supplements (*n* = 5), and omega-3 supplements (*n* = 12) that contained a nutrient of interest. If a reported supplement included a brand name and single nutrient, but no dose was recorded, then the supplement was identified as the lowest dose manufactured by that company.

Three sources of supplement label information were used to determine nutrient content: The Dietary Supplement Label Database (DSLD), 6 the Supplement Online Wellness Library (OWL) [28], and the brand or manufacturer’s website. Each unique supplement was first searched for in the DSLD and OWL. If the supplement was found in both of those sources and nutrient information matched, the DSLD information was downloaded for the database. If information on the DSLD and OWL differed, the most recently updated source was used. If a supplement was not found in the DSLD or OWL, the supplement label was downloaded from the supplement brand website. The Dietary Supplement Ingredient Database Release 4.0 (DSID-4) was developed and validated to improve the estimated nutrient intake from supplements. A wide variety of prenatal vitamins on the market were tested. The database predicts the mean difference in nutrient content between the value listed on a supplement label and the actual amount of nutrient contained in the supplement. A correction factor from the DSID was used in the analysis for the following vitamins and minerals: folic acid, niacin, riboflavin, thiamin, vitamin A, vitamin B6, vitamin B12, vitamin C, vitamin D, vitamin E, calcium, chromium, copper, iodine, iron, magnesium, manganese, potassium, selenium, and zinc [29,30,31]. The DSID currently does not have an adjustment for choline; however, because choline has become a nutrient of concern during pregnancy [32,33], intake was analyzed using the label value for this study.

Supplemental folate and folic acid were interpreted as 1 μg = 1 μg, despite known differences in bioavailability. Both the DHQ-II and NDSR provide the output of folate intake as dietary folate equivalents (DFE), accounting for differences in the bioavailability of folate consumed as naturally occurring food folate compared to synthetic folic acid added to fortified foods. Most supplements contain the synthetic form of folate, folic acid, while some prenatal vitamins do include at least a portion of the supplemental folate as the natural, active form 5-methyltetrahydrofolate. Many supplements contain a combination of preformed vitamin A or provitamin A carotenoids in varying proportions, which is not always reported on the supplement label. Supplemental vitamin A intake was also interpreted as 1 IU preformed vitamin A = 1 IU provitamin A carotenoids, despite differences in bioavailability. It was then converted to retinol activity equivalents (mcg RAE) using the conversion factor 1 IU = 0.3 mcg RAE [34]. Both the DHQ-II and NDSR provide total vitamin A activity from dietary intake in mcg RAE.

### 2.6. Statistical Analysis

IBM SPSS for Windows (Version 27.0) was used to summarize the population characteristics [35]. The Diet*Calc and NDSR software were used for obtaining the estimated nutrient intake from the diet, as previously mentioned. For subjects in which 24 h recalls were used to assess their diet, the average nutrient intake from at least two recalls was used as the final estimate of nutrient intake. The analysis of supplement intake was completed using SAS® software Version 9.4 (SAS Institute Inc.: Cary, CA, USA) [36].

## 3. Results

All but 16 women in this analysis (0.02%) reported taking a dietary supplement in some amount and for some time during pregnancy; however, only 86.7% took a prenatal micronutrient supplement (Table 2).

Regarding vitamins, vitamin C, niacin, riboflavin, vitamin B12, vitamin B6, and thiamin (the latter two only for Hispanic/Latina participants who completed 24 h recalls) had fewer than 25% of the participants below the EAR from the diet data. Mean dietary intakes of vitamin B6 and thiamin were below the EAR for 31 and 36%, respectively, of the participants who completed the DHQ-II and 17 and 13%, respectively, for those who completed the 24 h recalls. The dietary intake of vitamin D was below the EAR for over 85% of the participants. Dietary Vitamin E was below the EAR for over 70% of the participants, and dietary folate was below the EAR for more than half of the participants. Like thiamin and vitamin B6, participants who completed the 24 h recalls differed from those who completed the DHQ-II in being less likely to be below the EAR for choline, though both methods of assessment identified choline as a micronutrient of risk (DHQ-II, 84% below the AI; 24 h recalls, 58% below the AI). The addition of supplements to the diet effectively addressed the population deficits for all vitamins except choline (Table 3); however, it increased the proportion above the tolerable UL for folic acid to approximately 80%.

Regarding minerals, approximately 30% of the participants were below the EAR or AI for calcium, 33% for zinc, 40% for magnesium, 85% for iron, and 52.5% for potassium, while fewer than 10% of the women had dietary intakes below the EAR or AI for copper and selenium when their diet alone was considered (Table 4). Supplements corrected most of the population’s inadequate intake of iron and zinc but only half of the proportion with calcium below the AI. Supplementation did not address gaps in dietary intake for magnesium and potassium. Iodine and chromium are also minerals of interest in pregnancy.

While the dietary intake of chromium and iodine could not be determined, the amount of the AI contributed by supplements was small, suggesting that if these minerals are deficient in the diet, the deficiencies would not be corrected by prenatal supplements. A database for iodine in foods was published after the study was completed [37].

Of the 928 women with reliable data for supplement intake, over 90% reported taking a supplement that contained folic acid, niacin, vitamin A, vitamin B12, vitamin B6, vitamin C, vitamin D, and vitamin E, while fewer than 25% took a supplement containing choline, chromium, manganese, potassium, and selenium (Table 5).

Of the 136 unique prenatal multivitamin–mineral supplements consumed, more than 90% contained varying amounts of folic acid, niacin, vitamin A, vitamin B12, vitamin B6, vitamin C, vitamin D, vitamin E, and zinc and 75% or more contained calcium, iron, riboflavin, and thiamin. Fewer than 31% contained choline, chromium, manganese, potassium, or selenium (Table 6).

The trial randomized participants to a DHA supplement; therefore, their intake of a DHA supplement is not informative for US pregnant women. The DHA intake at the baseline may be related to that of other US cohorts. Their average intake of DHA from their diet was 84.5 mg (0–529 mg) and supplement intake averaged 84.4 mg (0–797 mg). Although 55.5% of the participants were taking a supplement of DHA, only 34.9% of those were taking a supplement of ≥200 mg, the amount recommended by several expert groups from seafood intake or supplements [1,38,39,40].

## 4. Discussion

Bailey et al. [7] and Jun et al. [3] assessed nutrient intake in pregnant women who participated in the National Health and Nutrition Examination Survey (NHANES) in the years 1999–2014, and a recent report from the 15 sites in the US ECHO consortium focused on the risk of inadequate and excessive intakes of micronutrients during pregnancy from 1999 to 2016 [8]. Like Bailey et al. [7] and the ECHO consortium [9], we wished to determine the effect of diet and the use of dietary supplements on micronutrient sufficiency and excess. Although our data were collected more recently, many of our findings are consistent with these reports.

A nutrient intake below the EAR is used to indicate the risk of deficiency in the population. For certain nutrients, an EAR is not established, and thus the AI was used as a comparison. Dietary intakes were below the EAR or AI for between 25 and 90% of the participants for vitamins A, D, and E, folate, choline, iron, calcium, magnesium, potassium, and zinc. Thiamin and vitamin B6 were also low among the participants who completed the DHQ-II. With the inclusion of dietary supplements, the intake of vitamins A, D, and E, folate, iron, and zinc improved, as did thiamin and vitamin B6, such that fewer than 10% of the participants had intakes below the EAR. In contrast, magnesium and potassium remained below the EAR or AI for between 24% and 84% of the participants and 16% below the AI for calcium. Supplementation increased folate intake above the UL for approximately 80% of the cohort and iron for 16.8%. Intakes above the UL for both micronutrients have been noted previously [3,7,8].

Only 16 women (0.02%) in the cohort did not take a dietary supplement at some time in the perinatal period, compared to 9.2% in the analysis of NHANES 1999–2014. Most dietary supplements consumed did not meet the definition of a prenatal dietary supplement, although most contributed at least one micronutrient for which the cohort had dietary insufficiency. Compared to results from NHANES, an identical proportion of the 1100 women enrolled in ADORE consumed a prenatal dietary supplement (80.5% vs. 80.4%) at some time during pregnancy [3,7]. We find, as did both NHANES [3] and the ECHO consortium [8], that the use of prenatal supplements is related to education and income. While the risk of inadequate intake of many micronutrients is improved by supplements, this is not necessarily the case for women who are underserved.

We report a higher percentage that exceeded the UL for folate (80%) than NHANES (33.4%) and ECHO (32–51%) [7,8]. Our values may be somewhat higher because we did not subtract natural food folate from folate-fortified grains; however, some of the differences may be due to the fact that data were collected after 2016 instead of from 1999. Even with folate fortification, dietary folate was below the EAR for nearly half of our cohort, evidencing a gap to be filled by dietary supplements; however, the amount of folate in many supplements could be reduced. It is interesting that the 24 h recall data of the Hispanic/Latina participants suggest that fewer may now consume an inadequate amount of dietary folate than the mostly non-Hispanic/Latina group who completed the DHQ-II (42% vs. 54%). Dietary folate intake among Hispanic/Latina women was a concern that led to the US Food and Drug Administration’s (FDA) approval of the fortification of corn masa flour on 14 April 2016. Our data suggest that the FDA policy of fortifying corn masa flour may have led to this level of folate adequacy seen among the Hispanic/Latina participants. However, more studies are needed.

Iron intakes were above the UL for 16.8% of the cohort, a value similar to Bailey et al. [7] (27.9%) but lower than Sauder et al. [8] (39–40%). Intakes above the UL with supplementation and the differences between our studies may reflect differences in the prescription of iron by caregivers to correct low blood hemoglobin.

Addressing deficiencies in magnesium, potassium, choline, and calcium with supplementation would likely require women to consume more than a single capsule as they are required in milligrams rather than the microgram amounts required for most vitamins. Vegetables and fruits are the best dietary sources of potassium; leafy green vegetables, whole grains, legumes, and nuts for magnesium; dairy products, fortified foods, and tofu for calcium [41]; and eggs for choline. Dietitians and others who care for pregnant women can educate pregnant women on how these categories of foods contribute to optimal nutrient intake in their pregnancy beyond a prenatal supplement rather than giving general advice to “consume a healthy diet.” The ECHO consortium found that achieving an intake of potassium above the AI was related to education and age and inversely related to BMI, [8] evidencing that lifestyle changes could be used to improve intake. The proportion of the cohort with an intake of potassium below the AI was 52.5%, analogous to both ECHO and NHANES. However, we find a smaller proportion of participants below the EAR for iron, vitamin D, and vitamin E than Bailey et al. [7] when diet and dietary supplements are combined. The differences between the studies may reflect a response by prenatal manufacturers to address dietary deficiencies that have been identified.

The first 1000 days of life from conception to 2 years is a critical period for micronutrients on lifelong health [4], and thus it is important to understand for pregnancy. When more than 50% of the population is below the EAR or AI, many individuals are likely deficient, but some individuals are likely deficient even when population studies suggest there is a low risk of inadequate intake, such as here for vitamins A and D. Both vitamin D [42] and vitamin A [43] deficiency are reported in pregnant women in the US, even though neither vitamin would be expected to be deficient in the population based on intake as reported here and by Bailey et al. [7].

A strength of the present study is the large, diverse cohort with good representation of racial and ethnic minorities and maternal education and income. Another strength is that we asked participants questions about supplement intake beginning before pregnancy and throughout pregnancy, including obtaining the label for the products used and carefully recording any changes to the product or usage throughout the period of interest, something that has not been conducted previously. By providing data for nutrient intake from the diet and supplements separately, this study identifies how to better target the micronutrient needs of US pregnant women. Lastly, using the DSID provides a better estimate of micronutrient intake from supplements, as the label frequently underestimates actual nutrient content.

A possible limitation of this study is that two different methods were used to collect dietary intake. The ECHO trial concluded there were differences between the recall and FFQ data. They reported results separately as we have here [8]. Nevertheless, both methods identified a similar proportion of participants with a dietary intake below the EAR or AI for vitamins A, D, and E, folate, iron, calcium, magnesium, potassium, and zinc. Choline was an exception as were niacin, thiamin, and vitamin B6. For each of these nutrients, the recall method identified fewer participants below the EAR or AI. Without a more detailed analysis of the diet, we cannot know if these differences are related to the method of collection or to differences in the nutrient content of the foods consumed by participants.

In summary, in the population of the US pregnant women we studied, prenatal supplements appear to fill much of the gap that exists between dietary intake and the DRI for iron and most vitamins without raising a significant concern for excessive micronutrient intake, with the exception of folate. At the same time, our data suggest that a high proportion of pregnant women have inadequate intakes of potassium and magnesium and, to a lesser extent, calcium. All three micronutrient minerals are associated with protection against eclampsia and preeclampsia [44,45,46]. Another micronutrient of concern is choline, a nutrient important to term delivery [47] and neural tube formation [48]. Pregnant women need clear directions on the importance of dietary supplements; however, they also need information from dietitians and other providers, as dietary supplements do not fill the gap for all micronutrients between diet and micronutrient requirements. To fill those gaps, pregnant women need to focus on consuming specific categories of foods. More research is needed across populations to examine the adequacy of intake in this critical time-period.

## Figures and Tables

**Table 1:** Description of the diverse population studied.

Baseline Characteristic	Total N = 1021	DHQ-II N = 843 (82.57%)	24 h Recalls N = 178 (17.43%)
Site			
Kansas City	450 (44.1%)	272 (32.3%)	178 (100.0%)
Columbus	348 (34.1%)	348 (41.3%)	0 (0.0%)
Cincinnati	223 (21.8%)	223 (26.5%)	0 (0.0%)
Language			
English	885 (86.7%)	842 (99.9%)	43 (24.2%)
Spanish	136 (13.3%)	1 (0.1%)	135 (75.8%)
Data Available			
Both dietary and supplement data	684 (67.0%)	561 (66.6%)	123 (69.1%)
Dietary intake data only	93 (9.1%)	81 (9.6%)	12 (6.7%)
Supplement intake data only	244 (23.9%)	201 (23.84%)	43 (24.16%)
Black/African American (yes/no) (including bi- and multiracial)			
No, not Black/African American	787 (77.1%)	611 (72.5%)	176 (98.9%)
Yes, Black/African American	233 (22.8%)	231 (27.4%)	2 (1.1%)
Unknown	1 (0.1%)	1 (0.12%)	0 (0.0%)
Race/Ethnicity			
American Indian or Alaskan Native	4 (0.5%)	4 (0.5%)	0 (0.0%)
Asian	25 (3.1%)	25 (3.1%)	0 (0.0%)
Bi- or Multiracial ^a^	22 (2.7%)	22 (2.7%)	0 (0.0%)
Black or African American	210 (26.0%)	210 (26.0%)	0 (0.0%)
Hispanic or Latina	211 (20.7%)	33 (3.9%)	178 (100.0%)
Native Hawaiian or Pacific Islander	1 (0.1%)	1 (0.1%)	0 (0.0%)
White	545 (67.5%)	545 (67.5%)	0 (0.0%)
Unknown	3 (0.3%)	3 (0.4%)	0 (0.0%)
Maternal Education (years)	14.6 ± 3.2	15.3 ± 2.8	11.3 ± 2.9
Paternal Education (years)	14.2 ± 3.2	14.8 ± 2.8	10.8 ± 3.0
Maternal Age at Enrollment (years)	30.4 ± 5.6	30.4 ± 5.5	30.2 ± 6.0
Parity (each)	1.2 ± 1.4	1.1 ± 1.4	1.7 ± 1.5
Gestational Age at Birth (weeks)	38.7 ± 1.9	38.7 ± 2.0	38.9 ± 1.4
# Supplements reported during pregnancy ^b^	2.4 ± 1.7	2.5 ± 1.8	2.0 ± 1.1

DHQ-II, diet history questionnaire II. Data are N (%) or Mean ± SD. ^a^ DHQ-II: Asian, Black (*n* = 1); Asian, Black, White (*n* = 1); Asian, White (*n* = 7); Black, Native American, White (*n* = 2); Black, White (*n* = 10); Native American, White (*n* = 1). ^b^ Data are from 928 women with reliable supplement intake information.

**Table 2:** Description of the use of a prenatal supplement in the entire ADORE study sample (*n* = 1100) by maternal language, race/ethnicity, family income, and education. Of the women who enrolled in this study, 80.5% reported taking a prenatal micronutrient supplement, and women who identified as Black/African American were less likely than women who identified as Hispanic/Latina and Non-Hispanic/Latina White to use a prenatal supplement.

	Total N	Yes PNV ^b^	No PNV ^b^
Total (entire ADORE cohort) ^a^	1100	885 (80.5%)	215 (19.5%)
Language			
English	940	762 (81.1%)	178 (18.9%)
Spanish	160	123 (76.9%)	37 (23.1%)
Race/Ethnicity			
American Indian or Alaskan Native	4	3 (75.0%)	1 (25.0%)
Asian	28	22 (78.6%)	6 (21.4%)
Bi- or Multiracial	27	23 (85.2%)	4 (14.8%)
Black/African American	267	187 (70.0%)	80 (30.0%)
Hispanic/Latina, any race	245	193 (78.8%)	52 (21.2%)
White	649	545 (84.0%)	104 (16.0%)
Income			
Less than USD 10,000	155	95 (61.3%)	60 (38.7%)
USD 10,000–USD 24,999	211	161 (76.3%)	50 (23.7%)
USD 25,000–USD 49,999	190	148 (77.9%)	42 (22.1%)
USD 50,000–USD 99,999	206	176 (85.4%)	30 (14.6%)
USD 100,000–USD 149,999	188	174 (92.6%)	14 (7.4%)
USD 150,000 or more	118	109 (92.4%)	9 (7.6%)
Unknown	32	22 (68.8%)	10 (31.3%)
Education			
Less than High School	159	119 (74.8%)	40 (25.2%)
High School Diploma/GED	233	161 (69.1%)	72 (30.9%)
Some college or tech school	213	164 (77.0%)	49 (23.0%)
Bachelor’s degree obtained	249	223 (89.6%)	26 (10.4%)
Master’s degree obtained	160	142 (88.8%)	18 (11.3%)
Doctorate	86	76 (88.4%)	10 (11.6%)
Maternal Age at Enrollment (years)	30.2 ± 5.6	30.5 ± 5.5	28.9 ± 6
Parity (each)	1.2 ± 1.4	1.1 ± 1.4	1.4 ± 1.3
Gestational Age at Birth (weeks)	38.7 ± 2	38.7 ± 1.9	38.5 ± 18

ADORE, The Assessment of DHA on Reducing Early Preterm Birth; PNV, prenatal vitamin. Data are N (%) or Mean ± SD. ^a^ Data from all 1100 participants of the ADORE trial. ^b^ PNV defined as having ≥3 vitamins + ≥1 mineral and specifically marketed for conception, prenatal, or postnatal use.

**Table 3:** The proportion of participants who met the EAR or AI or exceeded the UL for vitamin intake from diet alone or when supplements were included for both methods used to estimate dietary intake, while Table 4 is similar for mineral intake.

Nutrient	Diet Intake Method	Intake Type	N (%) < EAR or < AI ^a^	N (%) > UL ^b^	Median (IQR)	% Intake from Diet	% Intake from Supplements
Choline (mg) ^c^	DHQ-II	Diet + Supplement (*n* = 561)	470 (83.8%)	0 (0.0%)	304.5 (168.6)	98.7%	1.3%
		Diet alone (*n* = 642)	539 (84.0%)	0 (0.0%)	303.6 (176.6)	*	*
	24 h Recall	Diet + Supplement (*n* = 123)	73 (59.4%)	0 (0.0%)	417.0 (232.0)	99.7%	0.3%
		Diet alone (*n* = 135)	78 (57.8%)	0 (0.0%)	419.7 (234.4)	*	*
Folate (μg DFE) ^d^	DHQ-II	Diet + Supplement (*n* = 561)	11 (2.0%)	439 (78.3%)	1233.9 (416.0)	44.3%	55.7%
		Diet alone (*n* = 642)	347 (54.1%)	35 (5.5%)	497.2 (296.4)	*	*
	24 h Recall	Diet + Supplement (*n* = 123)	2 (1.6%)	104 (84.6%)	1249.1 (350.0)	49.7%	50.3%
		Diet alone (*n* = 135)	55 (40.7%)	12 (8.9%)	629.0 (367.9)	*	*
Niacin (mg) ^e^	DHQ-II	Diet + Supplement (*n* = 561)	6 (1.1%)	1.3%	37.0 (13.3)	58.0%	42.0%
		Diet alone (*n* = 642)	130 (20.3%)	*	20.4 (12.4)	*	*
	24 h Recall	Diet + Supplement (*n* = 123)	0 (0.0%)	0.6%	40.0 (11.0)	62.1%	37.9%
		Diet alone (*n* = 135)	6 (4.4%)	*	24.1 (11.7)	*	*
Riboflavin (mg)	DHQ-II	Diet + Supplement (*n* = 561)	9 (1.6%)	ND	3.7 (2.0)	63.3%	36.7%
		Diet alone (*n* = 642)	52 (8.1%)	ND	2.2 (1.2)	*	*
	24 h Recall	Diet + Supplement (*n* = 123)	0 (0%)	ND	3.5 (1.2)	63.5%	36.5%
		Diet alone (*n* = 135)	7 (5.2%)	ND	2.2 (1.0)	*	*
Thiamin (mg)	DHQ-II	Diet + Supplement (*n* = 561)	39 (7.0%)	ND	2.6 (1.3)	59.2%	40.8%
		Diet alone (*n* = 642)	232 (36.1%)	ND	1.4 (0.8)	*	*
	24 h Recall	Diet + Supplement (*n* = 123)	1 (0.8%)	ND	3.0 (1.0)	64.5%	35.5%
		Diet alone (*n* = 135)	17 (12.6%)	ND	1.8 (0.8)	*	*
Vitamin A (RAE) ^f^	DHQ-II	Diet + Supplement (*n* = 561)	11 (2.0%)	14 (2.5%)	1719.4 (711.5)	51.2%	48.8%
		Diet alone (*n* = 642)	170 (26.5%)	4 (0.6%)	771.1 (552.3)	*	*
	24 h Recall	Diet + Supplement (*n* = 123)	1 (0.8%)	16 (13.0%)	2195.3 (1113.2)	43.1%	56.9%
		Diet alone (*n* = 135)	32 (23.7%)	2 (1.5%)	843.3 (543.6)	*	*
Vitamin B12 (μg)	DHQ-II	Diet + Supplement (*n* = 561)	2 (0.4%)	ND	11.5 (6.1)	43.5%	56.5%
		Diet alone (*n* = 642)	59 (9.2%)	ND	4.6 (3.5)	*	*
	24 h Recall	Diet + Supplement (*n* = 123)	0 (0.0%)	ND	10.6 (4.0)	48.6%	51.4%
		Diet alone (*n* = 135)	12 (8.9%)	ND	5.1 (3.1)	*	*
Vitamin B6 (mg)	DHQ-II	Diet + Supplement (*n* = 561)	6 (1.1%)	0 (0.0%)	4.9 (5.9)	40.7%	59.3%
		Diet alone (*n* = 642)	199 (31.0%)	0 (0.0%)	2.0 (1.3)	*	*
	24 h Recall	Diet + Supplement (n = 123)	0 (0.0%)	2 (1.6%)	4.9 (1.6)	47.7%	52.3%
		Diet alone (*n* = 135)	23 (17.0%)	0 (0.0%)	2.3 (1.0)	*	*
Vitamin C (mg)	DHQ-II	Diet + Supplement (*n* = 561)	12 (2.1%)	0 (0.0%)	190.7 (114.1)	61.4%	38.6%
		Diet alone (*n* = 642)	145 (22.6%)	0 (0.0%)	110.5 (94.5)	*	*
	24 h Recall	Diet + Supplement (*n* = 123)	4 (3.3%)	0 (0.0%)	185.8 (117.7)	65.0%	35.0%
		Diet alone (*n* = 135)	27 (20.0%)	0 (0.0%)	121.3 (107.0)	*	*
Vitamin D (IU)	DHQ-II	Diet + Supplement (*n* = 561)	66 (11.8%)	13 (2.3%)	643.2 (453.6)	28.1%	71.9%
		Diet alone (*n* = 642)	584 (91.0%)	0 (0.0%)	151.0 (139.8)	*	*
	24 h Recall	Diet + Supplement (*n* = 123)	7 (5.7%)	0 (0.0%)	642.9 (234.9)	41.7%	58.3%
		Diet alone (*n* = 135)	116 (85.9%)	0 (0.0%)	261.8 (169.9)	*	*
Vitamin E (IU) ^g^	DHQ-II	Diet + Supplement (*n* = 561)	35 (6.24%)	0 (0.0%)	36.3 (14.6)	43.8%	56.2%
		Diet alone (*n* = 642)	452 (70.4%)	0 (0.0%)	13.4 (9.2)	*	*
	24 h Recall	Diet + Supplement (*n* = 123)	12 (9.8%)	0 (0.0%)	32.8 (13.3)	41.3%	58.7%
		Diet alone (*n* = 135)	111 (82.2%)	0 (0.0%)	11.7 (7.7)	*	*

AI, adequate intake; DFE, dietary folate equivalent; DHQ-II, diet history questionnaire-II; EAR, estimated average requirement; IQR, interquartile range; IU, international units; ND, not determinable; RAE, retinol activity equivalents; UL, tolerable upper-intake level. ^a^ N (%) = number (percentage) of participants <EAR or <AI. ^b^ N (%) = number (percentage) of participants >UL. ^c^ Adequate intake of choline. ^d^ Folate UL should only apply to synthetic forms obtained from supplements, fortified foods, or a combo or the two. This value includes some contribution of natural food folate from fortified foods and so overestimates the proportion above the UL. ^e^ Niacin UL only applies to synthetic forms obtained from supplements, fortified foods, or a combo or the two. ^f^ Vitamin A UL only applies to preformed vitamin A. Intake calculated including all forms. ^g^ Vitamin E UL only applies as a-tocopherol; applies to any form of supplemental a-tocopherol. * Not applicable.

**Table 4:** Nutrient intake of minerals from diet + supplements and diet alone.

Nutrient	Diet Intake Method	Intake Type	N (%) < EAR or < AI ^a^	N (%) >UL ^b^	Median (IQR)	% Intake from Diet	% Intake from Supplements
Calcium (mg)	DHQ-II	Diet + Supplement (*n* = 561)	84 (15.0%)	23 (4.1%)	1200.3 (656.5)	87.1%	12.9%
		Diet alone (*n* = 642)	175 (27.3%)	21 (3.3%)	1048.6 (647.9)	*	*
	24 h Recall	Diet + Supplement (*n* = 123)	24 (19.5%)	2 (1.6%)	1152.1 (543.4)	85.3%	14.7%
		Diet alone (*n* = 135)	39 (28.9%)	1 (0.7%)	1017.0 (551.9)	*	*
Copper (mg)	DHQ-II	Diet + Supplement (*n* = 561)	18 (3.2%)	0 (0.0%)	1.9 (1.4)	81.2%	18.8%
		Diet alone (*n* = 642)	42 (6.5%)	0 (0.0%)	1.4 (0.8)	*	*
	24 h Recall	Diet + Supplement (*n* = 123)	8 (6.5%)	1 (0.8%)	1.4 (0.8)	92.0%	8.0%
		Diet alone (*n* = 135)	14 (10.4%)	1 (0.7%)	1.3 (0.5)	*	*
Iron (mg)	DHQ-II	Diet + Supplement (*n* = 561)	114 (20.3%)	81 (14.4%)	35.4 (16.5)	49.2%	50.8%
		Diet alone (*n* = 642)	573 (89.3%)	0 (0.0%)	13.5 (7.6)	*	*
	24 h Recall	Diet + Supplement (*n* = 123)	8 (6.5%)	29 (23.6%)	38.8 (11.2)	46.2%	53.8%
		Diet alone (*n* = 135)	105 (77.8%)	2 (1.5%)	16.5 (9.3)	*	*
Magnesium (mg)	DHQ-II	Diet + Supplement (*n* = 561)	194 (34.6%)	**	333.4 (166.5)	93.5%	6.5%
		Diet alone (*n* = 642)	268 (41.7%)	**	309.6 (158.0)	*	*
	24 h Recall	Diet + Supplement (*n* = 123)	30 (24.4%)	**	392.9 (150.1)	90.8%	9.2%
		Diet alone (*n* = 135)	46 (34.1%)	**	341.7 (141.6)	*	*
Manganese (mg) ^c^	DHQ-II	Diet + Supplement (*n* = 561)	77 (13.7%)	2 (0.4%)	3.2 (1.9)	95.7%	4.3%
		Diet alone (*n* = 642)	106 (16.5%)	2 (0.3%)	3.1 (1.7)	*	*
	24 h Recall	Diet + Supplement (*n* = 123)	15 (12.2%)	0 (0.0%)	3.2 (1.6)	99.6%	0.4%
		Diet alone (*n* = 135)	18 (13.3%)	0 (0.0%)	3.2 (1.7)	*	*
Potassium (mg) ^c^	DHQ-II	Diet + Supplement (*n* = 561)	324 (57.8%)	ND	2751.9 (1336.4)	99.9%	0.1%
		Diet alone (*n* = 642)	365 (56.9%)	ND	2757.5 (1410.3)	*	*
	24 h Recall	Diet + Supplement (*n* = 123)	58 (47.2%)	ND	2930.9 (1103.9)	100.0%	0.0%
		Diet alone (*n* = 135)	62 (45.9%)	ND	2940.0 (1085.6)	*	*
Selenium (μg)	DHQ-II	Diet + Supplement (*n* = 561)	36 (6.4%)	0 (0.0%)	93.6 (58.3)	95.1%	4.9%
		Diet alone (*n* = 642)	52 (8.1%)	0 (0.0%)	89.0 (51.6)	*	*
	24 h Recall	Diet + Supplement (*n* = 123)	0 (0.0%)	0 (0.0%)	120.3 (54.4)	99.4%	0.6%
		Diet alone (*n* = 135)	0 (0%)	0 (0.0%)	118.0 (53.7)	*	*
Zinc (mg)	DHQ-II	Diet + Supplement (*n* = 561)	20 (3.57%)	26 (4.6%)	24.5 (14.0)	51.8%	48.2%
		Diet alone (*n* = 642)	252 (39.25%)	0 (0.0%)	10.6 (6.3)	*	*
	24 h Recall	Diet + Supplement (*n* = 123)	12 (9.76%)	2 (1.6%)	20.0 (18.1)	67.2%	32.8%
		Diet alone (*n* = 135)	38 (28.15%)	0 (0.0%)	12.0 (5.5)	*	*
Chromium (μg) ^c^	DHQ-II	Supplement alone (*n* = 762)	684 (89.76%)	ND	0.0 (0.0)	*	*
	24 h Recall	Supplement alone (*n* = 166)	163 (98.19%)	ND	0.0 (0.0)	*	*
Iodine (μg)	DHQ-II	Supplement alone (*n* = 762)	533 (69.95%)	0 (0.0%)	75.1 (168.2)	*	*
	24 h Recall	Supplement alone (*n* = 166)	134 (80.72%)	0 (0.0%)	123.5 (147.9)	*	*

AI, adequate intake; DHQ-II, diet history questionnaire-II; EAR, estimated average requirement; IQR, interquartile range; IU, international units; ND, not determinable; UL, tolerable upper-intake level. ^a^ N (%) = number (percentage) of participants <EAR or <AI. ^b^ N (%) = number (percentage) of participants >UL. ^c^ Adequate intake for chromium, manganese, and potassium. * Not applicable. ** Upper limit only applies to supplemental magnesium.

**Table 5:** Participants taking a supplement containing the nutrients of interest ^a^.

Nutrient	N	%
Calcium	791	85.2%
Choline	212	22.8%
Chromium	108	11.6%
Copper	387	41.7%
Folate	916	98.7%
Iodine	610	65.7%
Iron	802	86.4%
Magnesium	436	47.0%
Manganese	112	12.1%
Niacin	898	96.8%
Potassium	31	3.3%
Riboflavin	793	85.4%
Selenium	125	13.5%
Thiamin	793	85.4%
Vitamin A	894	96.3%
Vitamin B12	904	97.4%
Vitamin B6	912	98.3%
Vitamin C	907	97.7%
Vitamin D	912	98.3%
Vitamin E	905	97.5%
Zinc	831	89.6%

^a^ Data are from the 928 women with reliable information about supplement intake.

**Table 6:** Summary of nutrient values for prenatal vitamins only ^a^.

Nutrient	EAR or AI	UL	Unit	N (%) of PNVs	N PNVs Containing ≥UL	Overall Label AVG	Label AVG if >0	Label Max
Calcium	800	2500	mg	105 (77.2%)	0	139.5	180.7	1000
Choline	450 ^b^	3500	mg	42 (30.9%)	0	17.1	55.4	460
Chromium	30 ^b^	ND	μg	31 (22.8%)	n/a	23.1	101.2	300
Copper	0.8	10	mg	69 (50.7%)	0	0.8	1.6	3
Folic Acid	520	1000 ^c^	μg	135 (99.3%)	33 c	791.0	796.8	1600
Iodine	160	1100	μg	85 (62.5%)	0	105.1	168.1	290
Iron	22	45	mg	110 (80.9%)	7	22.9	28.3	91.5
Magnesium	290	350 ^d^	mg	65 (47.8%)	1	35.1	73.4	500
Manganese	2 ^b^	11	mg	36 (26.5%)	0	0.6	2.1	6
Niacin	14	35 ^e^	mg	129 (94.8%)	5	19.1	20.1	40
Potassium	2900 ^b^	ND	mg	9 (6.6%)	n/a	0.9	13.3	50
Riboflavin	1.2	ND	mg	109 (80.2%)	n/a	3.1	3.8	35
Selenium	49	400	μg	39 (28.7%)	0	23.1	80.6	225
Thiamin	1.2	ND	mg	109 (80.2%)	n/a	2.9	3.6	40
Vitamin A	1833	10000 ^f^	IU	124 (91.2%)	0	3237.6	3550.9	8000
Vitamin B12	2.2	ND	μg	134 (98.5%)	n/a	19.4	19.7	300
Vitamin B6	1.6	100	mg	134 (98.5%)	0	7.8	8.0	50
Vitamin C	70	2000	mg	135 (99.3%)	0	85.7	86.3	500
Vitamin D	400	4000	IU	134 (98.5%)	0	622.0	631.3	3000
Vitamin E	17.88	1490 ^g^	IU	133 (97.8%)	0	28.0	28.6	200
Zinc	9.5	40	mg	125 (91.9%)	0	13.8	15.1	32

^a^ Data are from 136 unique prenatal multivitamins, defined as having ≥3 vitamins + ≥1 mineral and specifically marketed for conception, prenatal, or postnatal use. AI, adequate intake; AVG, average; EAR, estimated average requirement; ND, not determinable; n/a, not applicable; PNV, prenatal vitamin; UL, tolerable upper-intake level; ^b^ adequate intake for choline, chromium, manganese, and potassium. ^c^ Folate UL only applies to synthetic forms obtained from supplements, fortified foods, or a combo or the two. Thirty-two out of 33 contain exactly 1000 μg, equal to the UL, according to the supplement label. ^d^ Upper limit only applies to supplemental magnesium. ^e^ Niacin UL only applies to synthetic forms obtained from supplements, fortified foods, or a combo or the two. ^f^ Vitamin A UL only applies to preformed vitamin A. ^g^ Vitamin E UL only applies as a-tocopherol; applies to any form of supplemental a-tocopherol.

## Abbreviations

ADORE	Assessment of DHA on Reducing Early Preterm Birth
AI	Adequate Intake
DHQ-II	Diet History Questionnaire-II
DFE	Dietary Folate Equivalents
DSID-3	Dietary Supplement Ingredient Database Release 3.0
DSLD	Dietary Supplement Label Database
DRIs	Dietary Reference Intakes
EAR	Estimated Average Requirement
FFQ	Food Frequency Questionnaire
NCC	Nutrition Coordinating Center
NCI	National Cancer Institute
NDSR	Nutrition Data System for Research
NICHD	National Institute of Child Health & Human Development
ODS	Office of Dietary Supplements
OWL	Online Wellness Library
UL	Tolerable Upper-Intake Level
US	United States
USDA	United States Department of Agriculture
